# Dopamine Release Neuroenergetics in Mouse Striatal Slices

**DOI:** 10.3390/ijms25094580

**Published:** 2024-04-23

**Authors:** Msema Msackyi, Yuanxin Chen, Wangchen Tsering, Ninghan Wang, Hui Zhang

**Affiliations:** 1 Department of Neuroscience, Thomas Jefferson University, Philadelphia, PA 19107, USA; msema.msackyi@students.jefferson.edu (M.M.); yuanxin.chen@uga.edu (Y.C.); wangchen.n@ufl.edu (W.T.); ninghan.wang@drexel.edu (N.W.); 2 Department of Physiology & Pharmacology, Center for Neurological Disease Research, The University of Georgia, 501 D.W. Brooks Drive, Athens, GA 30602, USA

**Keywords:** dopamine release, glycolysis, oxidative phosphorylation, Parkinson’s disease, mitochondria respiration, fast-scan cyclic voltammetry

## Abstract

Parkinson’s disease (PD) is the second most common neurodegenerative disorder. Dopamine (DA) neurons in the substantia nigra pars compacta, which have axonal projections to the dorsal striatum (dSTR), degenerate in PD. In contrast, DA neurons in the ventral tegmental area, with axonal projections to the ventral striatum, including the nucleus accumbens (NAcc) shell, are largely spared. This study aims to uncover the relative contributions of glycolysis and oxidative phosphorylation (OxPhos) to DA release in the striatum. We measured evoked DA release in mouse striatal brain slices using fast-scan cyclic voltammetry applied every two minutes. Blocking OxPhos resulted in a greater reduction in evoked DA release in the dSTR when compared to the NAcc shell, while blocking glycolysis caused a more significant decrease in evoked DA release in the NAcc shell than in the dSTR. Furthermore, when glycolysis was bypassed in favor of direct OxPhos, evoked DA release in the NAcc shell decreased by approximately 50% over 40 min, whereas evoked DA release in the dSTR was largely unaffected. These results demonstrate that the dSTR relies primarily on OxPhos for energy production to maintain evoked DA release, whereas the NAcc shell depends more on glycolysis. Consistently, two-photon imaging revealed higher oxidation levels of DA terminals in the dSTR than in the NAcc shell. Together, these findings partly explain the selective vulnerability of DA terminals in the dSTR to degeneration in PD.

## 1. Introduction

Energy metabolism is crucial for the functioning of the brain, which requires energy in the form of ATP to fuel the basic cellular functions, including maintaining ion gradients and cycling presynaptic synaptic vesicles [1] The major source of energy in the brain is glucose, which is broken down through glycolysis and oxidative phosphorylation (OxPhos) in order to produce ATP. Glycolysis, which occurs in the absence of oxygen, rapidly produces a net total of two ATP and two molecules of pyruvate from glucose. OxPhos, which occurs in mitochondria, is slower than glycolysis but has a much higher yield of ATP production. OxPhos produces 32 ATP per molecule of glucose, and contributes roughly 80% of energy in the brain [2]. The brain uses the majority of energy at the synapses and nodes of Ranvier, where mitochondria is abundant and the demand for energy is high.

The utilization of energy at the presynaptic compartments and energetic cost of presynaptic function has been of enormous interest [3]. Recent studies in the mouse calyx of Held [4] and in rat hippocampal cell culture [5] have attempted to parse the contribution of each of the energy production pathways, OxPhos and glycolysis, in glutamatergic neurons. Research conducted in the mouse calyx of Held concluded that glycolysis has a greater effect on basal glutamatergic transmission than OxPhos [4]. Research conducted in single neuron rat hippocampal cultures expanded on the findings of the previous study [5]. The authors concluded that either OxPhos or glycolysis is sufficient for glutamate transmission, However, OxPhos was of primary importance for high demand transmission. In the absence of glycolysis, monocarboxylates (e.g., lactate) act as an alternative fuel source for oxidative phosphorylation. As anaerobic glycolysis occurs in astrocytes, lactate builds up and is subsequently shuttled to neurons through monocarboxylate transport (MCT) to be used for fuel [6,7]. Although energy production in the brain is well understood, the dependence of DA release on the different energy production systems remains unclear.

DA neurons are tonically active with extensive arborization [8], and they are thought to be more metabolically demanding than most neuron types in the brain [9]. This high metabolic demand could result in higher rates of energy production and oxidative stress. Cell culture research has shown that, when compared to the ventral tegmental area (VTA) neurons, SNc DA neurons have a higher basal rate of mitochondrial OxPhos, a lower reserve capacity of spare ATP, a higher density of axonal mitochondria and increased oxidative stress, and a considerably more complex axonal arborization [10]. However, to date, no one has studied the energetic cost of presynaptic DA neurotransmission. Studying the neuroenergetics of DA neurotransmission is particularly important in the context of neurodegenerative disorders, such as Parkinson’s disease (PD), which is characterized by mitochondrial dysfunction and oxidative stress [11,12]. In PD, the DA terminals in the dSTR projected from SNc DA neurons degenerate, whereas the DA terminals in the ventral striatum, including the NAcc shell projected from VTA DA neurons, are largely spared. In this study, we investigated the role of glycolysis and OxPhos in energy production for the evoked release of DA in the dSTR and the NAcc shell. We found that the dSTR relies more heavily on OxPhos for evoked DA release compared to the NAcc shell, while, in contrast, the NAcc shell relies more on glycolysis for evoked DA release compared to the dSTR.

## 2. Results

### 2.1. Experimental Design

The slices were stimulated every 2 min either in the dSTR or NAcc shell for 40 min, and DA release was measured using fast-scan cyclic voltammetry (FSCV). Single-pulse stimulation was employed to mitigate potential indirect effects from other neurotransmitters released during train stimulation. Moreover, given the significant probability of DA release in striatal slices, train stimulation does not induce much additional DA release. Pharmacological reagents selectively inhibiting glycolysis or OxPhos were added onto the mouse brain slices through perfusion, with an exchange time of 4 min in our system. Typically, electrically evoked DA release is highest on the first stimulation, then decreases to a stable baseline with subsequent stimulations. Experiments were generally initiated following three stimuli to ensure a consistent and robust response. Normalization was performed in comparison to the average of the first three stimuli, unless otherwise specified.

**Figure 1:**
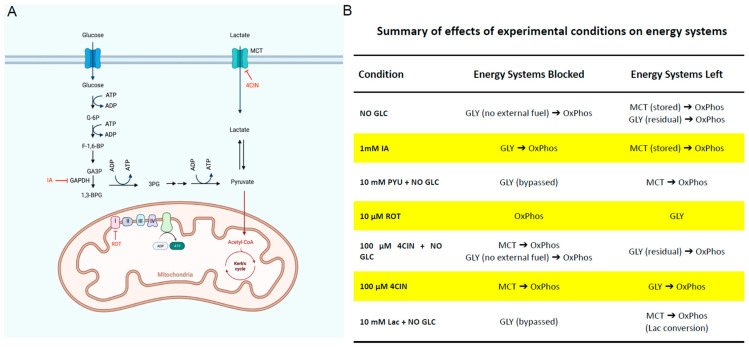
**Schematics and table showing cellular energy production systems under various conditions**. (**A**), A schematic showing the different energy systems in the striatum with inhibitors to block the different energy production pathways. (**B**), A table showing how the different conditions affect the various energy production systems associated with evoked DA release. 2DG—2 deoxy-D-glucose, GLC—glucose, P—phosphate, ROT—rotenone, Lac—lactate, IA—iodoacetic Acid, Pyu—pyruvate, 4CIN—α-Cyano-4-hydroxycinnamic acid, Gly—glycolysis, MCT—monocarboxylate transport, OxPhos—oxidative phosphorylation.

Energy production systems under various conditions are shown in the schematic and table (Figure 1). Notably, although OxPhos produces roughly 16 times more ATP than glycolysis produces per glucose molecule, the process is much more time consuming than glycolysis. OxPhos has many more steps than glycolysis, including two whole turns of the eight-step Krebs cycle per glucose molecule, followed by ATP production via the mitochondria complex and the electron transport chain (Figure 1A). The MCT system itself does not produce energy; it transports mainly lactate as fuel for OxPhos (Figure 1A). The pathway leading from the import of glucose or the breakdown of glycogen to pyruvate, and then to lactate to be exported is the pathway astrocytes use for MCT [7]. 

### 2.2. Evoked DA Release Is Higher in the dSTR than in the NAcc Shell

**Figure 2:**
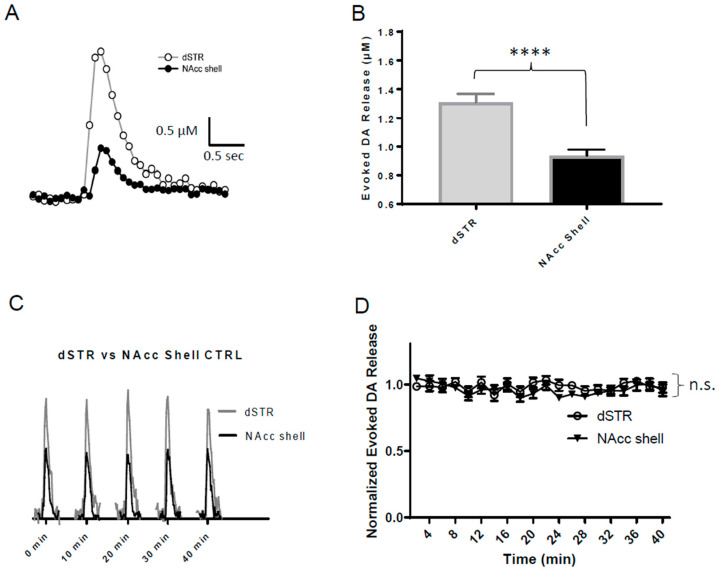
**Evoked DA release is higher in the dSTR than in the NAcc shell.** Evoked DA release in the dSTR is significantly higher than that in the NAcc shell. (**A**), Representative FSCV traces of 1p-stimulation-evoked DA release recorded in the dSTR and the NAcc shell. (**B**), Plot showing average amplitudes of 1p-evoked DA release (dSTR, *n* = 8; NAcc shell, *n* = 9 NAcc shell, **** *p* < 0.0001, Student *t*-test). (**C**), Representative traces of 1p-evoked DA release over 40 min taken every 10 min under control conditions. (**D**), Normalized evoked DA release over 40 min measured every 2 min in the dSTR and the NAcc shell (dSTR, *n* = 6; NAcc shell, *n* = 6, n.s., two-way ANOVA with Bonferroni post hoc test).

To investigate the evoked DA release in the dSTR and the NAcc shell, we measured the DA release via a single-pulse electrical stimulus every 2 min for 40 min under regular glucose-containing ACSF using FSCV (Figure 2A). Consistent with previous findings, the evoked DA release in the dSTR was significantly higher than that in the NAcc shell (Figure 2B,C), with a dSTR:NAcc shell ratio for DA release of 1:0.71 (Figure 2C). The evoked DA release in both the dSTR and the NAcc shell is stable over 40 min, showing no significant differences with an insignificant 5% run-down (Figure 2D).

### 2.3. Similar Diminishment of Evoked DA Release in the dSTR and the NAcc Shell under Glucose-Deprived Conditions

**Figure 3:**
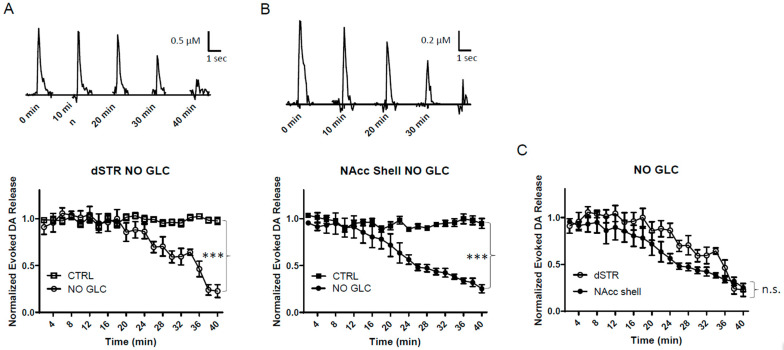
**Similar diminishment of evoked DA release in the dSTR and the NAcc shell under glucose-deprived conditions.** (**A**), Representative traces of 1p-evoked DA release in the dSTR over 40 min taken every 10 min under glucose-deprived conditions (NO GLC, top) and plot of the normalized evoked DA release over 40 min measured every 2 min under glucose-deprived conditions (bottom, NO GLC, *n* = 6; control (CTRL), *n* = 6, NO GLC vs. CTRL, *** *p* < 0.001, two-way ANOVA with Bonferroni post hoc test). (**B**), Representative traces of 1p-evoked DA release in the NAcc shell over 40 min taken every 10 min under glucose-deprived conditions (top) and the plot of the normalized evoked DA release over 40 min measured every 2 min under glucose-deprived conditions (bottom, NO GLC, *n* = 6; CTRL, *n* = 6, NO GLC vs. CTRL, *** *p* < 0.001, two-way ANOVA with Bonferroni post hoc test). (**C**), Comparison of normalized evoked DA release in the dSTR and NAcc shell over 40 min under glucose-deprived conditions showing no statistical significance, dSTR vs. NAcc shell, n.s., two-way ANOVA with Bonferroni post hoc test.

To test the evoked DA release in the dSTR relative to the NAcc shell under conditions of energy deprivation, we perfused striatal slices with ACFS containing no glucose. Under the conditions of glucose deprivation, the evoked DA release in both areas decreased over time with a significant diminishment compared to their respective controls (Figure 3A,B). The diminishment of the evoked DA release in the dSTR was similar to the NAcc shell (Figure 3C). Under these conditions, no external fuel was perfused in the slices that could be used for energy production.

### 2.4. Glycolysis Inhibition Diminishes Evoked DA Release in the NAcc Shell More Rapidly than in the dSTR

**Figure 4:**
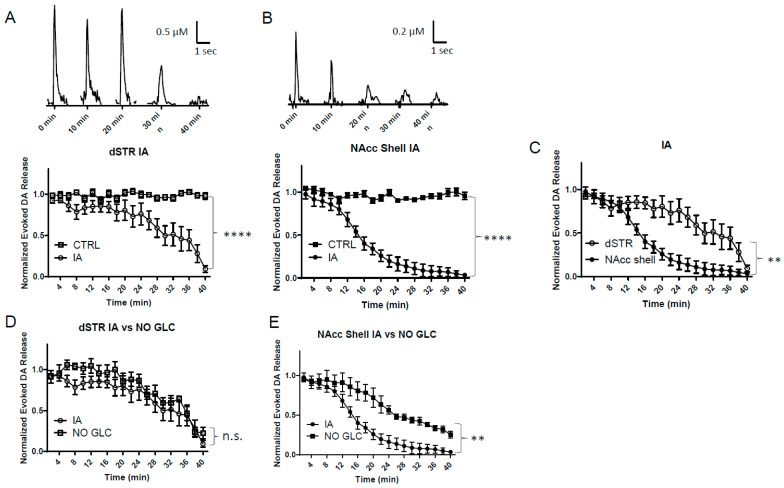
**Glycolysis inhibition diminishes evoked DA release in the NAcc shell more rapidly than in the dSTR.** (**A**), Representative traces of 1p-evoked DA release in the dSTR over 40 min taken every 10 min when glycolysis was inhibited with 1 mM IA (top) and a plot of the normalized evoked DA release over 40 min measured every 2 min (bottom, IA, *n* = 6; CTRL, *n* = 6, IA vs. CTRL, **** *p* < 0.0001, two-way ANOVA with Bonferroni post hoc test). (**B**), Representative traces of 1p-evoked DA release in the NAcc over 40 min taken every 10 min when glycolysis was inhibited with 1 mM IA (top) and a plot of the normalized evoked DA release over 40 min measured every 2 min (bottom, IA, *n* = 6; CTRL, *n* = 6, IA vs. CTRL, **** *p* < 0.0001, two-way ANOVA with Bonferroni post hoc test). (**C**), Comparison of normalized evoked DA release in the dSTR and NAcc shell over 40 min under glycolysis inhibition conditions, dSTR vs. NAcc shell, ** *p* < 0.01, two-way ANOVA with Bonferroni post hoc test. (**D**), Comparison of normalized evoked DA release in the dSTR over 40 min under glycolysis inhibition and glucose-deprived conditions, showing no significant difference, IA, *n* = 6; NO GLC, *n* = 6, IA vs. NO GLC, ** *p* < 0.01, n.s., two-way ANOVA with Bonferroni post hoc test. (**E**), Comparison of normalized evoked DA release in the NAcc over 40 min under glycolysis inhibition and glucose-deprived conditions, showing glycolysis inhibition diminishes evoked DA release more rapidly than glucose-deprived conditions, IA, *n* = 6; NO GLC, *n* = 6, IA vs. NO GLC, ** *p* < 0.01, two-way ANOVA with Bonferroni post hoc test.

We then perfused the brain slices with glycolysis inhibitor iodoacetate (IA, 1 mM) to investigate the importance of glycolytic energy production for evoked DA release in the striatum. IA blocks glycolysis by inhibiting the conversion of glyceraldehyde-3-phosphate (GA3P) to 1,3-bisphosphoglycerate (1,3-BPG). (Figure 1A). IA was used under regular ACFS conditions. Under these conditions, evoked DA in the dSTR and the NAcc shell diminished by approximately 90% over 40 min (Figure 4A,B). The diminishment in the evoked DA release occurred more rapidly in the NAcc shell when compared to the dSTR (Figure 4C). This indicates that, while glucose is present, the NAcc shell relies more heavily on glucose than the dSTR does for evoked DA release. Evoked DA release in the dSTR was similar in this condition when compared to glucose-deprived conditions over 40 min perfusion (Figure 4D); however, evoked DA release in the NAcc shell was significantly lower in this condition than under glucose-deprived conditions (Figure 4E). This suggests that the presence of glucose is associated with an increased dependence on glycolysis for evoked DA release in the NAcc shell. In contrast, evoked DA release in the dSTR is similarly dependent on glycolysis, regardless of whether glucose is present or not. Thus, the dSTR relies more on non-glycolytic energy than the NAcc shell when glucose is present.

### 2.5. Bypassing Glycolysis Diminishes Evoked DA Release in the NAcc Shell More Rapidly than the dSTR

**Figure 5:**
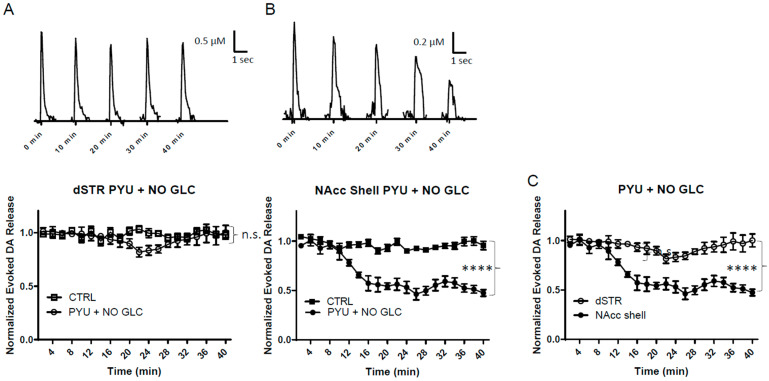
**Bypassing glycolysis diminishes evoked release of DA in the NAcc shell more than in the dSTR.** (**A**), Representative traces of 1p-evoked DA release in the dSTR over 40 min taken every 10 min under when 10 mM pyruvate was administered under glucose-deprived conditions (PYU + NO GLC, top) and a plot of the normalized evoked DA release over 40 min measured every 2 min (bottom, PYU + NO GLC, *n* = 5; CTRL; *n* = 6, PYU + NO GLC vs. CTRL, n.s., two-way ANOVA with Bonferroni post hoc test). (**B**), Representative traces of 1p-evoked DA release in the NAcc shell over 40 min taken every 10 min under glucose-deprived conditions (top) and a plot of the normalized evoked DA release over 40 min measured every 2 min (bottom, PYU + NO GLC, *n* = 5; CTRL, *n* = 6, PYU + NO GLC vs. CTRL, **** *p* < 0.0001, two-way ANOVA with Bonferroni post hoc test). (**C**), Comparison of normalized evoked DA release in the dSTR and NAcc shell over 40 min under condition bypassing glycolysis, dSTR vs. NAcc shell, **** *p* < 0.0001, two-way ANOVA with Bonferroni post hoc test.

At the end of glycolysis, glucose is converted into two molecules of pyruvate, which feed the Krebs cycle and lead to OxPhos (Figure 1A). Pyruvate is therefore important for OxPhos, and can bypass glycolysis to produce ATP. This is important in determining how well OxPhos alone can maintain evoked DA release. Under glucose-deprived conditions, 10 mM pyruvate was perfused and the evoked DA release in the dSTR was measured. The evoked DA release was slightly diminished for the first 20 min, then it rebounded, and by the end of the perfusion, the evoked DA release was the same as the control condition (Figure 5A). This initial diminishment could be caused by glycolysis being bypassed, hence the quick production of ATP from glycolysis would be abrogated. This dynamism did not occur for the evoked DA release in the NAcc shell. The evoked release in the NAcc shell kept decreasing, and was diminished by roughly 50% over 40 min (Figure 5B). Aside from an initial diminishment, the evoked DA release in the dSTR was maintained at the control levels, whereas the evoked DA release in the NAcc shell dropped to half of the control levels (Figure 5C). This indicates that the evoked DA release in the dSTR is more reliant on OxPhos than the NAcc shell.

### 2.6. Specific Inhibition of OxPhos Diminishes Evoked DA Release in the dSTR More than the NAcc Shell

**Figure 6:**
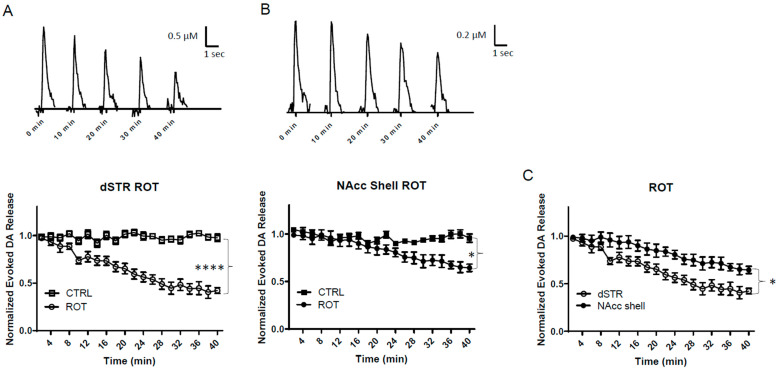
**Specific inhibition of OxPhos diminishes evoked release of DA in the dSTR more than the NAcc shell.** (**A**), Representative traces of 1p-evoked DA release in the dSTR over 40 min taken every 10 min when complex I inhibited with 10 µM rotenone (ROT, top) and a plot of the normalized evoked DA release over 40 min measured every 2 min (bottom, ROT, *n* = 5; CTRL, *n* = 6, ROT vs. CTRL, **** *p* < 0.0001, two-way ANOVA with Bonferroni post hoc test). (**B**), Representative traces of 1p-evoked DA release in the NAcc shell over 40 min taken every 10 min when complex I inhibited with 10 µM ROT (top) and a plot of the normalized evoked DA release over 40 min measured every 2 min (bottom, ROT, *n* = 5; CTRL, *n* = 6, ROT vs. CTRL, * *p* < 0.05, two-way ANOVA with Bonferroni post hoc test). (**C**), Comparison of normalized evoked DA release in the dSTR and NAcc shell over 40 min under specific inhibition of OxPhos, dSTR vs. NAcc shell, * *p* < 0.05, two-way ANOVA with Bonferroni post hoc test.

We then assessed the effect of direct inhibiting OxPhos on the evoked DA release in these brain areas through the use of rotenone (ROT), an inhibitor of complex I of the mitochondrial electron transport chain (Figure 1A). When 10 μM ROT was applied to the brain slices, the evoked DA release in both the dSTR and the NAcc shell was significantly diminished (Figure 6A,B), but the diminishment in the dSTR was quicker and significantly greater than that in the NAcc shell (~55% vs. ~30%, * *p* < 0.05, Figure 6C). Together with the condition of bypassing glycolysis using pyruvate (Figure 5C), these results suggest that the dSTR is more reliant than NAcc on OxPhos. Furthermore, these results, in combination with the results from Figure 4C, indicate that the NAcc is more reliant than dSTR on glycolysis for DA release.

### 2.7. MCT Inhibition Reduces Evoked DA Release in the dSTR but Not in the NAcc Shell

**Figure 7:**
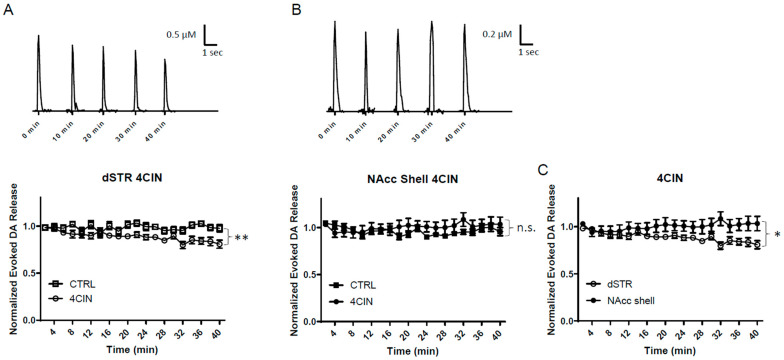
**MCT inhibition reduces evoked DA release in the dSTR but not in the NAcc shell.** (**A**), Representative traces of 1p-evoked DA release in the dSTR over 40 min taken every 10 min when MCT inhibited with 100 µM 4CIN (top) and a plot of the normalized evoked DA release over 40 min measured every 2 min (bottom, 4CIN, *n* = 6; CTRL, *n* = 6, 4CIN vs. CTRL, ** *p* < 0.01, two-way ANOVA with Bonferroni post hoc test). (**B**), Representative traces of 1p-evoked DA release in the NAcc shell over 40 min taken every 10 min MCT inhibited with 100 µM 4CIN (top) and a plot of the normalized evoked DA release over 40 min measured every 2 min (bottom, 4CIN, *n* = 6; CTRL, *n* = 6, 4CIN vs. CTRL, n.s., two-way ANOVA with Bonferroni post hoc test). (**C**), Comparison of normalized evoked DA release in the dSTR and NAcc shell over 40 min under inhibition of MCT, dSTR vs. NAcc shell, * *p* < 0.05, two-way ANOVA with Bonferroni post hoc test.

The MCT system may play a significant role in energy production, especially under conditions of high energy demands, which increase glucose consumption, mostly in astrocytes [13,14,15]. Astrocytes mainly fuel neurons via glucose metabolites, such as lactate or pyruvate (Lac: PYU = 10:1), which are then shuttled via MCTs to neighboring neurons [6,7]. To examine the role of the MCT system and their relative importance to energy production in DA terminals, slices were perfused with 100 μM α-cyano-4-hydroxycinnamic acid (4CIN), a non-specific inhibitor of the MCT system (Figure 1A). When 4CIN was administered under regular glucose conditions, the evoked DA release in the dSTR was slightly but significantly diminished as compared to the control (Figure 7A). This suggests that under normal conditions, dSTR utilizes the MCT system as a fuel for energy production but not at a high level. In contrast, there was no significant diminishment of evoked DA release in the NAcc shell (Figure 7B). There was an initial decrease in the evoked DA release in the NAcc shell, but the diminishment was recovered over 40 min of perfusion. Interestingly, the evoked DA release in the NAcc shell became highly variable during the course. Since glycolytic cells produce a high level of lactate that is exported under normal conditions, this variability in the evoked DA release could be due to a buildup of lactate waste since the export of lactate was blocked, leading to acidification. In summary, the MCT system fuels evoked DA release in the dSTR slightly, but not in the NAcc shell when glucose is present (Figure 7C).

### 2.8. MCT Inhibition under Glucose-Deprived Conditions Reduces Evoked DA Release in the Striatum More Rapidly Compared to Glucose-Deprived Conditions

**Figure 8:**
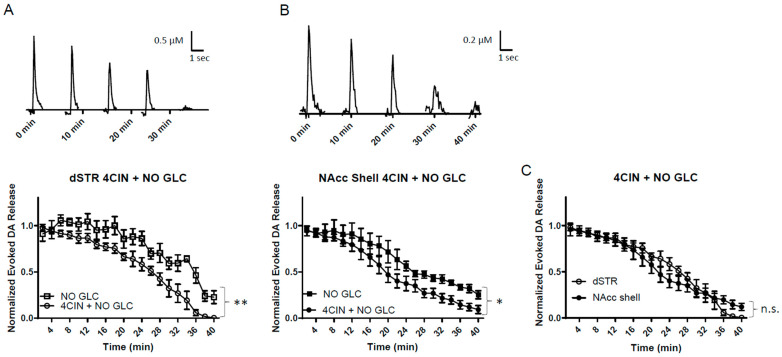
MCT inhibition under glucose-deprived conditions reduces evoked DA release in the striatum more rapidly compared to glucose-deprived conditions. (**A**), Representative traces of 1p-evoked DA release in the dSTR over 40 min taken every 10 min when MCT inhibited with 100 µM 4CIN under glucose-deprived conditions (top) and a plot of the normalized evoked DA release over 40 min measured every 2 min (bottom, 4CIN + NO GLC, *n* = 6; NO GLC, *n* = 6, 4CIN + NO GLC vs. NO GLC, ** *p* < 0.01, two-way ANOVA with Bonferroni post hoc test). (**B**), Representative traces of 1p-evoked DA release in the dSTR over 40 min taken every 10 min when MCT inhibited with 100 µM 4CIN under glucose-deprived conditions (top) and a plot of the normalized evoked DA release over 40 min measured every 2 min (bottom, 4CIN + NO GLC, *n* = 6; NO GLC, *n* = 6, 4CIN + NO GLC vs. NO GLC, * *p* < 0.05, two-way ANOVA with Bonferroni post hoc test). (**C**), Comparison of normalized evoked DA release in the dSTR and NAcc shell over 40 min under MCT inhibition under glucose-deprived conditions, dSTR vs. NAcc shell, n.s., two-way ANOVA with Bonferroni post hoc test.

To investigate the MCT system under energy-deprived conditions, slices were perfused with 100 μM 4CIN under glucose-deprived conditions. There is evidence that energy deprivation increases MCT from astrocytes [7,13,16]. Under glucose-deprived conditions, the evoked DA release diminished more rapidly when compared to glucose deprivation alone in both regions (Figure 8A,B). The diminishment of the evoked DA release in the dSTR and the NAcc shell was similar in response to 4CIN, with no significant difference in these two areas (Figure 8C). This suggests that both the dSTR and the NAcc shell rely on energy produced by the MCT system in the absence of glucose.

### 2.9. Energy Production via the MCT System Maintains Evoked DA Release at a Higher Level in the dSTR than in the NAcc Shell

**Figure 9:**
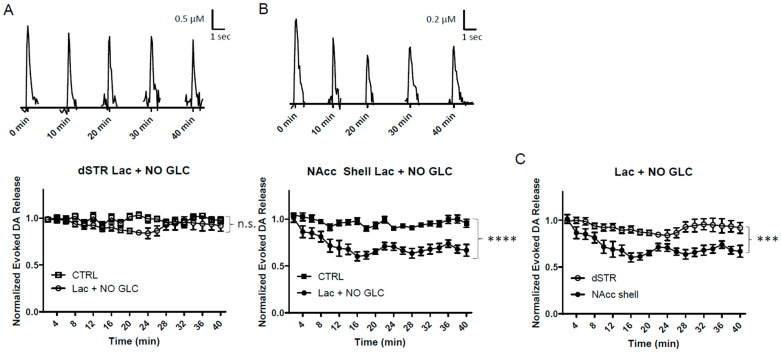
**Energy production via the MCT system can maintain evoked DA release in the dSTR but not in the NAcc shell**. (**A**), Representative traces of 1p-evoked DA release in the dSTR over 40 min taken every 10 min when 10 mM lactate was administered under glucose-deprived condition (Lac + NO GLC, top) and a plot of the normalized evoked DA release over 40 min measured every 2 min (bottom, Lac + NO GLC, *n* = 6; CTRL, *n* = 6, Lac + NO GLC vs. CTRL, n.s., two-way ANOVA with Bonferroni post hoc test). (**B**), Representative traces of 1p-evoked DA release in the NAcc shell over 40 min taken every 10 min when 10 mM lactate was administered under glucose-deprived condition (Lac + NO GLC, top) and a plot of the normalized evoked DA release over 40 min measured every 2 min (bottom, Lac + NO GLC, *n* = 6; CTRL, *n* = 6, Lac + NO GLC vs. CTRL, **** *p* < 0.0001, two-way ANOVA with Bonferroni post hoc test). (**C**), Comparison of normalized evoked DA release in the dSTR and NAcc shell over 40 min when 10 mM lactate was administered under glucose-deprived condition, dSTR vs. NAcc shell, *** *p* < 0.001, two-way ANOVA with Bonferroni post hoc test.

Considering that lactate is the main glucose metabolite shuttled through the MCT system to neurons [6,7], the slices were perfused with 10 mM lactate under glucose-deprived conditions. This can serve as another measurement of OxPhos energy production fueled by MCT system. This condition is complementary to the measuring of the impact of inhibiting MCT on the evoked DA release (Figure 8). Lactate can be used as fuel for OxPhos after it is converted to pyruvate (Figure 1A). Lactate, when perfused under glucose-deprived conditions, led to an initial suppression of evoked DA release in the dSTR (Figure 9A), but this diminishment largely rebounded over 40 min of perfusion. In contrast, the diminishment was more drastic in the NAcc shell and did not rebound (Figure 9B). The evoked DA release was diminished to a greater degree in the NAcc shell when compared to the dSTR (Figure 9C). The lactate and pyruvate conditions were similar, but the initial diminishment in the evoked DA release in the dSTR was longer with lactate, possibly due to the conversion of lactate to pyruvate which can then fuel OxPhos.

### 2.10. Higher Oxidation Level in the DA Terminals in the dSTR than That in the NAcc

**Figure 10:**
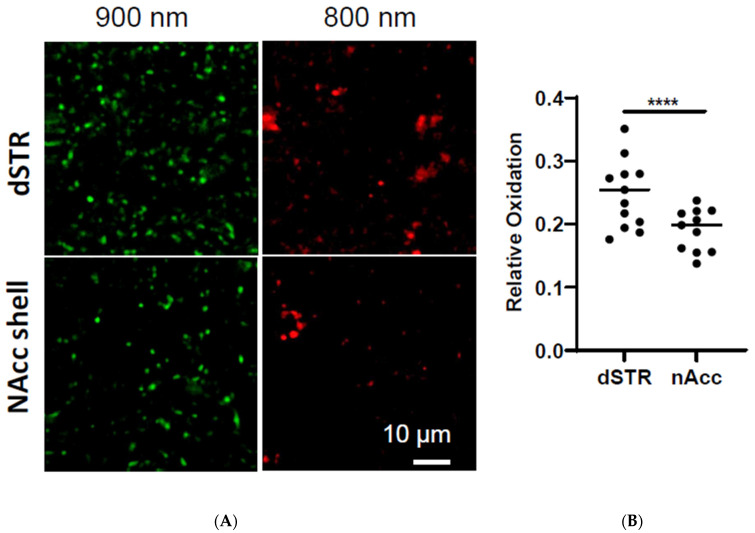
**Oxidation level is higher in the dSTR than in the NAcc shell**. (**A**), Representative images of roEGFP signals in the dSTR and NAcc excited at 900 nm and 800 nm. (**B**), Plot of average oxidation level (800/900) in the dSTR (11 slices) and the NAcc (11 slices) from four mice aged 4–7 months old, dSTR vs. NAcc, **** *p* < 0.0001, Student *t*-test.

The results present here show that, in general, the evoked DA release in the dSTR largely depends on OxPhos energy production, while the evoked DA release in the NAcc shell depends more on glycolytic energy production. The byproducts of OxPhos are reactive species, such as reactive oxygen species, that can lead to oxidative stress [12]. To directly assess whether there is a higher oxidation level in the dSTR, we measured the oxidation level using two-photon imaging of TH-mito-roGFP mice [17]. As expected, the oxidation level was higher in the dSTR than in the NAcc (Figure 10).

## 3. Discussion

A large body of evidence from PD research has shown that mitochondrial dysfunction and oxidative stress are associated with PD [12,17]. Previous research has shown that basal OxPhos is higher in cultured SNc DA neurons when compared to VTA DA neurons [10]. Here, we report that the evoked DA release in the dSTR is more dependent on OxPhos, while the evoked DA release in the NAcc shell is relatively more dependent on glycolysis. Consistent with the previous studies [10,17], our results demonstrate an overall tendency for DA cell bodies and neurites associated with PD to heavily rely on mitochondrial OxPhos. However, for the first time, we show a relative glycolytic preference for DA release in the NAcc shell.

### 3.1. Dorsal Striatum Prefers Oxidative Phosphorylation Energy Production for Evoked DA Release

The evoked DA release in the dSTR shows a clear preference for OxPhos for ATP production, while the evoked DA release in the NAcc shell shows a higher preference for glycolytic ATP production when compared to the dSTR. The glycolytic preference for ATP production in the NAcc shell was apparent both when glycolysis was blocked (Figure 4C) and when OxPhos was inhibited (Figure 6C). The evoked DA release in the NAcc shell was maintained by OxPhos at around 50% when compared to control conditions (Figure 5B, Figure 6B and Figure 9B). Under these conditions, the NAcc shell did not show a clear preference for either glycolysis or OxPhos for ATP production. Evoked release in the dSTR, however, clearly showed a preference for oxidative ATP production when OxPhos was selectively activated (Figure 5A and Figure 9A) and when OxPhos was inhibited (Figure 6A).

Under glucose-deprived conditions, although the overall evoked DA release in the dSTR and the NAcc shell diminished to a similar degree over 40 min, the DA release in the NAcc shell was not diminished for the first ~8 min compared to that in the dSTR (Figure 3C). This trend was abolished when the glycolysis inhibitor was administered (Figure 4E), suggesting that this is probably due to residual glycolysis.

### 3.2. Monocarboxylate Transport in the Striatum

Lactate is one type of monocarboxylate which must be exported out of cells, as high levels of intracellular accumulation of lactate result in the inhibition of glycolysis. However, some tissues, such as heart and brain, utilize lactate for cellular respiration, therefore serving as an attractive candidate for alternative metabolic substrates during glycolytic inhibition.

Under normal conditions, when glucose is present, the evoked DA release appears to be partially fueled by MCT in the dSTR, but not the NAcc shell (Figure 7 and Figure 9). This conclusion is further supported by the results of experiments with lactate in glucose-deprived conditions. Just as with pyruvate in glucose-deprived conditions (Figure 5C), the evoked DA release in the dSTR was significantly greater than the evoked DA release in the NAcc shell (Figure 9C). Moreover, the trends of the evoked DA release seen with lactate in glucose-deprived conditions are complementary with pyruvate in glucose-deprived conditions also (Figure 5 and Figure 9). This demonstrates that the NAcc shell is not as effective and efficient as the dSTR when using lactate or pyruvate for a fuel source.

Under glucose-deprived conditions, it seems that both the dSTR and the NAcc shell utilize lactate as a fuel to a similar extent (Figure 9C). Recent evidence has shown that lactate shuttling involves astrocytes, especially for axonal metabolism when energy demands are high or glucose levels are low in the brain [7,16]. Applying 4CIN in a glucose-deprived environment revealed a similar dependence on MCT to fuel the evoked DA release in both the dSTR and the NAcc shell, consistent with the previous evidence. This evidence in conjunction with Figure 8 and Figure 4 also suggests that the evoked DA release is more energetically demanding in the dSTR than the NAcc shell when glucose is present.

One limitation of this study is the possibility of indirect effects on DA terminals. While variations in the metabolic inhibitor’s impact on the evoked DA release in the dSTR and NAcc shell are evident, attributing the differences solely to nigral versus VTA innervation is unwarranted. The metabolic inhibitors tested will have effects on striatal neurons and glia that regulate axonal DA release, as well as on DA terminals. Indirect metabolic inhibition consequences on DA release via other striatal cells were not taken into account in the current experimental design. Thus, regional metabolic sensitivity differences may arise from other striatal cells, such as cholinergic interneuron influences, rather than directly affecting DA axons. Future experiments should address these factors in order to clarify regional metabolic sensitivity disparities stemming from cholinergic interneuron effects or direct DA axon impact.

### 3.3. Implications and Further Direction

The preference for oxidative energy production in the dSTR may be due to the overall difference in DA terminal density and complexity between the dSTR and the NAcc shell [8,10]. The higher release of DA in the dSTR is due to a higher DA terminal density and a greater terminal complexity in the dSTR when compared to the NAcc shell. Due to these factors, DA release in the dSTR is more energetically demanding than in the NAcc shell. To meet this increase in energy demand, energy production efficiency must be increased. Although both glycolysis and OxPhos produce ATP, ATP production via OxPhos is much more efficient than ATP production via glycolysis; increasing oxidative ATP production can significantly increase the overall energy production without significantly increasing glucose consumption. Therefore, increasing OxPhos relative to glycolysis supports DA release in the dSTR without increasing glucose consumption. A heavy reliance on OxPhos also allows the dSTR to be more flexible in its sources of fuel, since OxPhos can be also fueled by lactate or ketones. This flexibility would allow DA release in the dSTR to be fueled even if energy demands increase or fasting/starvation occurs. According to the results presented here, the MCT system can fuel evoked DA release in the dSTR at similar levels to glucose, allowing DA release in the dSTR to be higher and more stable than in the NAcc shell when provided with the same amounts of fuel.

The high dependence of dSTR on OxPhos could, in part, help to explain the specific vulnerability of the DA terminals in the dSTR to degeneration in PD when compared to the DA terminals in the NAcc shell. The dSTR utilizes OxPhos preferentially, and the byproducts of OxPhos are reactive oxygen species that can lead to oxidative stress [11,12]. Because the NAcc shell prefers glycolytic ATP production relatively, lower levels of oxidative stress and neurodegeneration would occur. But the protection may go further than that. The NAcc shell utilizes a relatively higher level of glycolysis to fuel the evoked DA release. Lactate, a byproduct of glycolysis, can activate the pathways associated with cellular survival, homeostasis, angiogenesis, and overall neuroprotection [7,12,15,18]. The upregulation of glycolysis is neuroprotective in a Drosophila model of amyotrophic lateral sclerosis [19]. Likewise, recent research in clinical models has shown that enhancing glycolysis attenuates PD symptoms [20]. Further studies examining the different expression levels of the metabolic genes and proteins may uncover the underlying mechanisms driving the different preferences of the ATP-producing pathways in the dSTR and the NAcc shell.

## 4. Materials and Methods

### 4.1. Animals and Slice Preparation

The use of the animals followed the National Institutes of Health guidelines and was approved by the Institutional Animal Care and Use Committee at Thomas Jefferson University and The University of Georgia. All efforts were made to minimize the number of animals used. Wildtype (WT) adult male mice aged between 4 and 10 months were used. TH-mito-roGFP mice expressing a redox-sensitive variant of green fluorescent protein (roGFP) with a mitochondrial-matrix targeting sequence under the control of the TH promoter were obtained from Dr. Surmeier at Northwestern University [17].

Mice were decapitated after cervical dislocation and their brains were immediately dissected out in a cold pre-oxygenated cutting solution (in mM: 125 NaCl, 2.5 KCl, 26 NaHCO_3_, 3.7 MgSO_4_,0.3 KH_2_PO_4_, 10 glucose, pH 7.4). Coronal striatal slices were sectioned with a vibrating microtome (VT1200, Leica, Solms, Germany) at a thickness of 300 μm and recovered in oxygenated artificial cerebrospinal fluid (ACSF, in mM, 125 NaCl, 2.5 KCl, 26 NaHCO_3_, 2.4 CaCl_2_, 1.3 MgSO_4_, 0.3 KH_2_PO_4_, 10 glucose, 2 HEPES, pH 7.4) at room temperature (RT) for at least 1 h. The brain slice was subsequently positioned within a recording chamber superfused (1.5 mL/min) with ACSF at 36 °C. At least 3 mice were used per condition, unless otherwise specified. All the chemicals were purchased from Sigma. We adjusted the osmolality as necessary, such as in the case of ACSF lacking glucose, by supplementing it with NaCl.

### 4.2. Fast-Scan Cyclic Voltammetry and Amperometry

Fast-scan cyclic voltammetry (FSCV) was used to measure the evoked DA release in the striatum. Electrochemical recording and electrical stimulations were performed as previously described [21]. Briefly, disk carbon fiber electrodes of 5 μm diameters with a freshly cut surface were placed into the dSTR or the NAcc shell ~50 μm below the exposed surface. For FSCV, a triangular voltage wave (−450 to +800 mV at 280 V/sec vs. Ag/AgCl) was applied to the recording electrode every 100 ms with a pulse duration of 8.5 ms and a ramp of 294 mV/ms via an Axopatch 200B (Axon Instruments, Foster City, CA, USA). The current was recorded with an Axopatch 200B amplifier with a low-pass Bessel filter setting at 10 kHz, digitized at 25 kHz (ITC-18 board; InstruTech, Great Neck, NY, USA). Triangular wave generation and data acquisition were controlled by a personal computer running a locally written (Dr. E. Mosharov, Columbia University, New York, NY, USA) IGOR program (WaveMetrics, Lake Oswego, OR, USA). Striatal slices were electrically stimulated by an Iso-Flex stimulus isolator, triggered by a Master-8 pulse generator (A.M.P.I., Jerusalem, Israel) using a bipolar stimulating electrode placed at a distance of ~100 μm from the recording electrode. Background-subtracted cyclic voltammograms were used for electrode calibration and to identify the released substance.

### 4.3. 2PLSM: Mitochondrial roEGFP Imaging in Living Brain

Striatal slices from TH-mito-roGFP male transgenic mice were imaged at physiological temperatures (36 °C). Optical imaging of roGFP signals were acquired using an Ultima multiphoton laser scanner (Bruker, Madison, WI, USA) for BX51/61 Olympus microscope with a Titanium-sapphire laser (Chameleon-Ultra2, Coherent, Inc., Santa Clara, CA, USA), equipped with a 20 × 1.0 NA water immersion objective (XLUMPLFL20XW, Olympus, Shinjuku City, Tokyo). Dual excitation beam (800 nm/900 nm) was used to excite roGFP protein, respectively, and roGFP fluorescence was collected at 490–560 nm. The ratio of 800 nm/900 nm was taken as an index of the oxidation level. Images were captured in 8-bit, 45 × 45 µm regions of interest at 512 × 512 pixels.

RoGFP exhibits two excitation peaks that respond to changes in redox states. The redox status was evaluated by monitoring the ratio of GFP fluorescence emission under excitation at 800 and 900 nm. For the assessment of the mitochondrial redox status in dopaminergic terminals, regions of interest (ROIs) were delineated around the terminals, as well as in background areas outside the cells. Pixel intensity was quantified from images captured at two wavelengths (800 and 900 nm), with background subtraction applied. The fluorescent intensity ratios were computed by dividing the 800 nm image by the 900 nm image on a pixel-by-pixel basis.

All multi-photon images were processed and analyzed using customized MATLAB scripts (Matlab 2020a, MathWorks, Natick, MA, USA).

### 4.4. Statistical Analysis

Experimental values in the text and in the figures are mean ± SEM. The Student’s *t*-test was used for paired data and two-way ANOVA, followed by Bonferroni post-test being performed using GraphPad Prism 8.0 (GraphPad software, San Diego, CA) to determine the statistical significance for all grouped data, unless otherwise specified. The difference was considered significant at levels of * *p* < 0.05; ** *p* < 0.01; *** *p* < 0.001; **** *p* < 0.0001; n.s. stands for no significance, and *n* stands for the number of experiments. Please refer to the Supplementary Table for detailed statistical information.

## Data Availability

Data supporting the findings of this study are available in this manuscript and Supplementary Materials. All other data that support the findings of this study are available from the corresponding authors on reasonable request.

## Supplementary Materials

The following supporting information can be downloaded at https://www.mdpi.com/article/10.3390/ijms25094580/s1.
