# Supplementary material for: Dopamine Release Neuroenergetics in Mouse Striatal Slices
Source: Int J Mol Sci. 2024 Apr 23;25(9):4580. doi: 10.3390/ijms25094580 (PMC11083938; doi:10.3390/ijms25094580)
Supplement: Supplementary file 1 [file ijms-25-04580-s001.zip › ijms-2923391-supplementary.pdf]

Supplementary Tabel S1

Statistics about two-way ANOVA

Figure	ANOVA Table	n	F	P
2D – dSTR CTRL vs NAcc shell CTRL	Time x condition	6,6	(19, 190) = 0.9466	0.5265
	Time (min)		(4.257, 42.57) = 1.841	0.1351
	Condition		(1, 10) = 1.369	0.2692
3A – dSTR NO GLC vs CTRL	Time x condition	6,6	(19, 190) = 10.26	<0.0001
	Time (min)		(3.735, 37.35) = 10.20	<0.0001
	Condition		(1, 10) = 28.69	0.0003
3B – NAcc shell NO GLC vs CTRL	Time x condition	6,6	F (19, 190) = 12.13	<0.0001
	Time (min)		F (19, 190) = 14.29	<0.0001
	Condition		F (1, 10) = 35.10	0.0001
3C – dSTR vs NAcc shell NO GLC	Time x condition	6,6	F (19, 190) = 1.118	0.3356
	Time (min)		F (19, 190) = 26.03	<0.0001
	Condition		F (1, 10) = 4.775	0.0538
4A – dSTR IA vs CTRL	Time x condition	6,6	F (19, 190) = 5.128	<0.0001
	Time (min)		F (19, 190) = 5.100	<0.0001
	Condition		F (1, 10) = 60.09	<0.0001
4B – NAcc shell IA vs CTRL	Time x condition	6,6	F (19, 190) = 38.48	<0.0001
	Time (min)		F (3.751, 37.51) = 47.60	<0.0001
	Condition		F (1, 10) = 124.0	<0.0001
4C – dSTR IA vs NAcc shell IA	Time x condition	6,6	F (19, 190) = 4.666	<0.0001
	Time (min)		F (19, 190) = 25.82	<0.0001
	Condition		F (1, 10) = 21.90	0.0009
4D – dSTR IA vs NO GLC	Time x condition	6,6	F (19, 190) = 0.4566	0.9758
	Time (min)		F (3.435, 34.35) = 15.77	<0.0001
	Condition		F (1, 10) = 4.328	0.0642
4E – NAcc IA vs NO GLC	Time x condition	6,6	F (19, 190) = 3.785	<0.0001
	Time (min)		F (3.295, 32.95) = 55.82	<0.0001
	Condition		F (1, 10) = 14.88	0.0032
5A – dSTR PYU + NO GLC vs CTRL	Time x condition	5,6	F (19, 171) = 1.577	0.0668
	Time (min)		F (3.980, 35.82) = 1.213	0.3223
	Condition		F (19, 171) = 1.577	0.1183
5B – NAcc shell PYU + NO GLC vs CTRL	Time x condition	5,6	F (19, 171) = 9.092	<0.0001
	Time (min)		F (19, 171) = 14.05	<0.0001
	Condition		F (1, 9) = 181.5	<0.0001
5C– dSTR vs NAcc shell PYU + NO GLC	Time x condition	5,5	F (19, 152) = 5.022	<0.0001
	Time (min)		F (19, 152) = 10.39	<0.0001
	Condition		F (1, 8) = 107.8	<0.0001
6A – dSTR ROT vs CTRL	Time x condition	5,6	F (19, 171) = 15.21	<0.0001
	Time (min)		F (4.668, 42.01) = 15.39	<0.0001
	Condition		F (1, 9) = 82.27	<0.0001
6B – NAcc shell ROT vs CTRL	Time x condition	5,6	F (19, 171) = 6.943	<0.0001
	Time (min)		F (19, 171) = 9.689	<0.0001
	Condition		F (1, 9) = 8.664	0.0164

6C– dSTR vs NAcc shell ROT	Time x condition	5,5	F (19, 152) = 2.464	0.0013
	Time (min)		F (19, 152) = 39.56	<0.0001
	Condition		F (1, 8) = 10.09	0.0131
7A – dSTR 4CIN vs CTRL	Time x condition	6,6	F (19, 190) = 1.857	0.0195
	Time (min)		F (4.781, 47.81) = 1.768	0.1403
	Condition		F (1, 10) = 20.09	0.0012
7B – NAcc shell 4CIN vs CTRL	Time x condition	6,6	F (19, 190) = 0.9709	0.4972
	Time (min)		F (2.649, 26.49) = 0.8159	0.4834
	Condition		F (1, 10) = 0.8536	0.3773
7C – dSTR vs NAcc shell 4CIN	Time x condition	6,6	F (19, 190) = 1.794	0.0258
	Time (min)		F (2.660, 26.60) = 0.4456	0.6998
	Condition		F (1, 10) = 6.439	0.0295
8A – dSTR 4CIN + NO GLC vs NO GLC	Time x condition	6,6	F (19, 190) = 1.903	0.0158
	Time (min)		F (4.320, 43.20) = 46.47	<0.0001
	Condition		F (1, 10) = 16.03	0.0025
8B – NAcc shell 4CIN + NO GLC vs NO GLC	Time x condition	6,6	F (19, 190) = 0.8208	0.6806
	Time (min)		F (19, 190) = 45.73	<0.0001
	Condition		F (1, 10) = 5.051	0.0484
8C– dSTR vs NAcc shell 4CIN + NO GLC	Condition	6,6	F (19, 190) = 2.275	0.0027
	Time (min)		F (19, 190) = 89.01	<0.0001
	Time x condition		F (1, 10) = 1.434	0.2588
9A– dSTR Lac + NO GLC vs CTRL	Time x condition	6,6	F (19, 190) = 1.377	0.1421
	Time (min)		F (3.290, 32.90) = 1.127	0.3552
	Condition		F (1, 10) = 3.539	0.0893
9B – NAcc shell Lac + NO GLC vs CTRL	Time x condition	6,6	F (19, 190) = 1.849	0.0202
	Time (min)		F (2.878, 28.78) = 5.639	0.0040
	Condition		F (1, 10) = 84.08	<0.0001
9C– dSTR vs NAcc shell Lac + NO GLC	Time x condition	6,6	F (19, 190) = 1.697	0.0395
	Time (min)		F (19, 190) = 4.859	<0.0001
	Condition		F (1, 10) = 28.15	0.0003
